# Mandibular characteristics of early Glires (Mammalia) reveal mixed rodent and lagomorph morphotypes

**DOI:** 10.1098/rstb.2022.0087

**Published:** 2023-07-03

**Authors:** Łucja Fostowicz-Frelik, Philip G. Cox, Qian Li

**Affiliations:** ^1^ Department of Organismal Biology and Anatomy, University of Chicago, Chicago, IL 60637, USA; ^2^ Institute of Paleobiology, Polish Academy of Sciences, 00-818 Warsaw, Poland; ^3^ Centre for Integrative Anatomy, Department of Cell and Developmental Biology, University College London, London WC1E 6BT, UK; ^4^ Key Laboratory of Evolutionary Systematics of Vertebrates, Institute of Vertebrate Paleontology and Paleoanthropology, Chinese Academy of Sciences, Beijing 100044, People's Republic of China; ^5^ Center for Excellence in Life and Paleoenvironment, Chinese Academy of Sciences, Beijing 100044, People's Republic of China

**Keywords:** Glires, Lagomorpha, Rodentia, Palaeocene, mandible, morphology

## Abstract

Glires (rodents, lagomorphs and their fossil kin) is the most speciose and arguably most diversified clade of living placentals. Different lineages within the Glires evolved basically opposite chewing movements: a mostly transversal power stroke in lagomorphs, and a mostly proal power stroke in rodents, but the ancestral condition for Glires is still unclear. To address this knowledge gap, we studied the mandibles of Chinese Palaeocene Glires representing the duplicidentate (lagomorph-like; *Mimotona*) and simplicidentate (rodent-like; *Eomylus* and *Heomys*) lineages. To assess the mechanical resistance of mandibles to bending and torsion, we calculated the section modulus. The dentaries differ greatly in morphology and the region where the maximum grinding force was likely applied. The early Palaeocene *Mimotona lii* and the middle Palaeocene *Mimotona robusta* and *Heomys orientalis* all show a pattern of increasing strength moving posteriorly along the mandible, similar to sciurids and the mountain beaver. By contrast, the late Palaeocene *Eomylus* sp. mandible was strongest in the m1 region, a pattern seen in lagomorphs and the stem placental *Zofialestes*. Our results indicate the early diversification of mandible structure of Glires, demonstrate a mixture of duplicidentate and simplicidentate characters among the basal Glires and suggest an early occurrence of a lagomorph-like morphotype.

This article is part of the theme issue ‘The mammalian skull: development, structure and function’.

## 1. Introduction

With over 2600 recognized species [1], Glires are the most numerous and diverse group of living mammals. Morphologically, Glires are defined primarily by a unique set of mandibular and dental characters including enlarged ever-growing incisors, loss of canines and some reductions in the premolar dentition [2], which creates a toothless diastema separating two functionally decoupled regions in the jaws responsible for different stages of food acquisition and processing [3].

Modern Glires consist of two clades, Lagomorpha and Rodentia, recognized as sister taxa [2]. Of the two, lagomorphs are conservative in many features, including body size, dentition, the foot, skull, and brain structure [4–8], and have an unusually uniform body plan, which appears only in two varieties: short-limbed ochotonids (pikas) and long-limbed leporids (rabbits and hares). Rodents, on the other hand, span a wide range of body sizes (particularly when fossil taxa are considered e.g. [9]) and have a skull shape notably distinct from other mammals [10]. The oldest (mostly Palaeocene) Glires belonging to the stem [11,12] cannot be unequivocally ascribed to either rodents or lagomorphs, but their morphology is somewhat intermediate between both groups [2,11,13–16]. The stem taxa tentatively associated with the origins of Lagomorpha have been placed in Duplicidentata (e.g. Mimotonidae), and those regarded as closer to Rodentia in Simplicidentata (e.g. Eurymylidae) [11,12,17].

The mandible is a particularly important element for understanding ecology in fossil taxa as its function is almost exclusively related to feeding. Various methodological approaches investigating the link between form and function have been applied to the mandibles of Glires (mostly rodents), including geometric morphometrics (e.g. [18–20]), estimation of jaw muscle mechanical advantages (e.g. [21–23]) and finite-element analysis (e.g. [24,25]). Here, we used cantilever beam theory to determine mandibular resistance to bending and torsion via analysis of cross-sectional geometry. Previous research has demonstrated a relationship between mandibular cross-sectional geometry and dietary specialization in extant carnivorans [26] and bats [27] and has been used to infer feeding ecology in extinct reptiles and mammals [28–30].

In this paper we analyse the morphological features of the mandible in the earliest stem Glires representatives from the early to late Palaeocene of China. We aimed to establish, whether the mandible structure of these basal forms more closely resembles the lagomorph-like or rodent-like morphotype. For the first time we use micro-CT data to analyse the mandibular mechanical properties, namely the cross-sectional strength, of these unique specimens. Furthermore, the archaic Glires morphotypes are compared with the Cretaceous eutherian *Zofialestes* of an important family of stem placental mammals (zalambdalestids; see [31]), whose mandible is strikingly similar to that of Glires in some respects [32], and which sometimes is considered close to Glires origins ([33]; see also [32]).

Finally, we compared these mandibles with a sample of extant and fossil lagomorphs and rodents; the latter comprising taxa associated with sciuromorph, hystricomorph, protrogomorph and (pseudo)myomorph morphotypes [34].

## 2. Material and methods

### (a) Sample

Although dentaries are relatively common remains of the Palaeogene Glires (e.g. [12,15,35,36]), complete mandibles are extremely rare, and none is known for the Palaeocene taxa. There are no intact angular or condylar processes preserved, and in most of these specimens the diastemal part is also partly destroyed. Complete mandibles of stem Glires have been reported only for the early Eocene duplicidentate *Gomphos elkema* from Mongolia [11] and the eurymylid *Rhombomylus turpanensis* from China [2]; both species represent later and more specialized Glires lineages.

The material used for the study consists of mandible bodies (here, ‘dentaries’) with the cheek teeth, and incisor fragments. The fossil Glires material comprises *Heomys orientalis* (IVPP V 4321, a right dentary with p4–m3 and the roots of p3 and lower incisor (di2?)), *Mimotona lii* (IVPP V 4327, a right dentary with p3–m3) and *Mimotona robusta* (IVPP V 4329, a right dentary with p4–m2). These unique specimens represent the oldest Glires fossils from the early to middle Palaeocene of the Qianshan locality, Anhui Province, China [12,15,37,38]. *Eomylus* sp. (IVPP V 25372, a right dentary with p4–m3 and roots of p3 and lower incisor (di2?)) complements this sample as a late Palaeocene (lower Nomogen Formation, NM-1) representative of stem Glires from the Nuhetingboerhe locality, Nei Mongol, China [39]. Our fossil Glires sample also includes the classic North American stem lagomorph *Palaeolagus haydeni* (see [40,41]). We used the zalambdalestid *Zofialestes longidens* (ZPAL MgM-I/135, a right dentary with p3–m3, roots of c and p1, and a lower incisor) from the late Cretaceous of Mongolia as a model for possible Glires ancestor within a stem placental morphotype.

**Figure 1.**
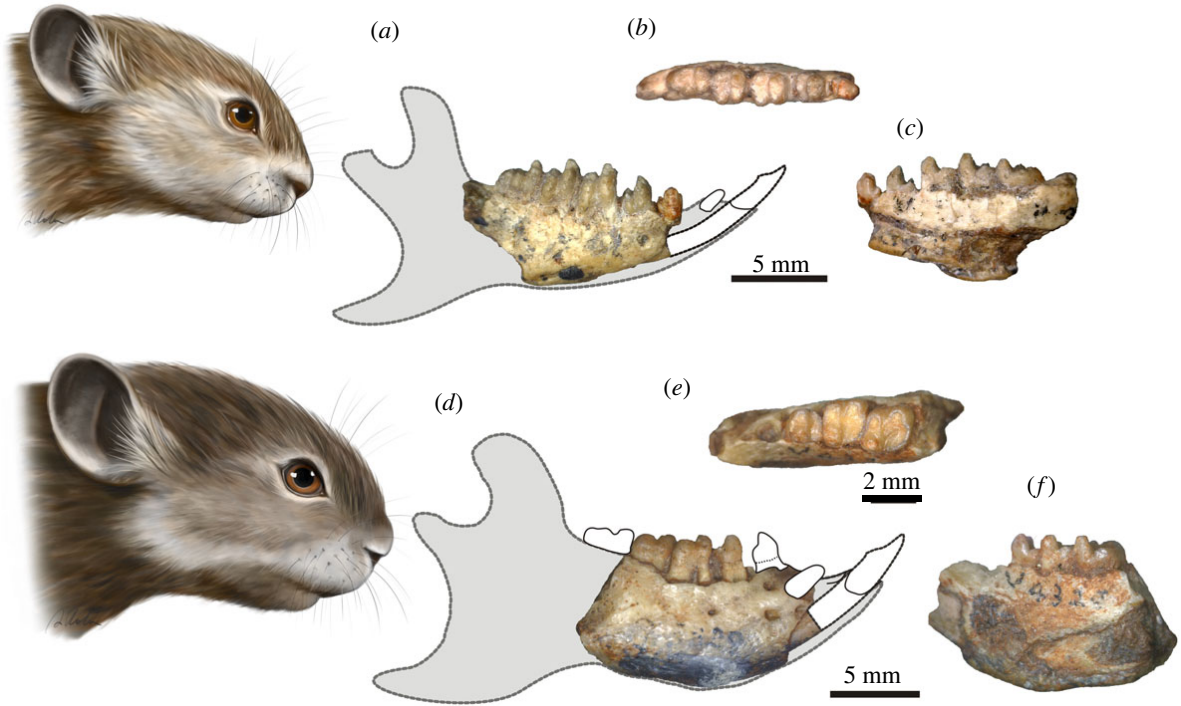
Mandible morphology in earliest representatives of duplicidentate Glires. (*a–c*) *Mimotona lii* (IVPP V 4327) in buccal, occlusal and lingual views. (*d–f*) *Mimotona robusta* (IVPP V 4329) in buccal, occlusal and lingual views; both Qianshan locality, Anhui Province, China. Head visualizations by Agnieszka Kapuścińska.

**Figure 2.**
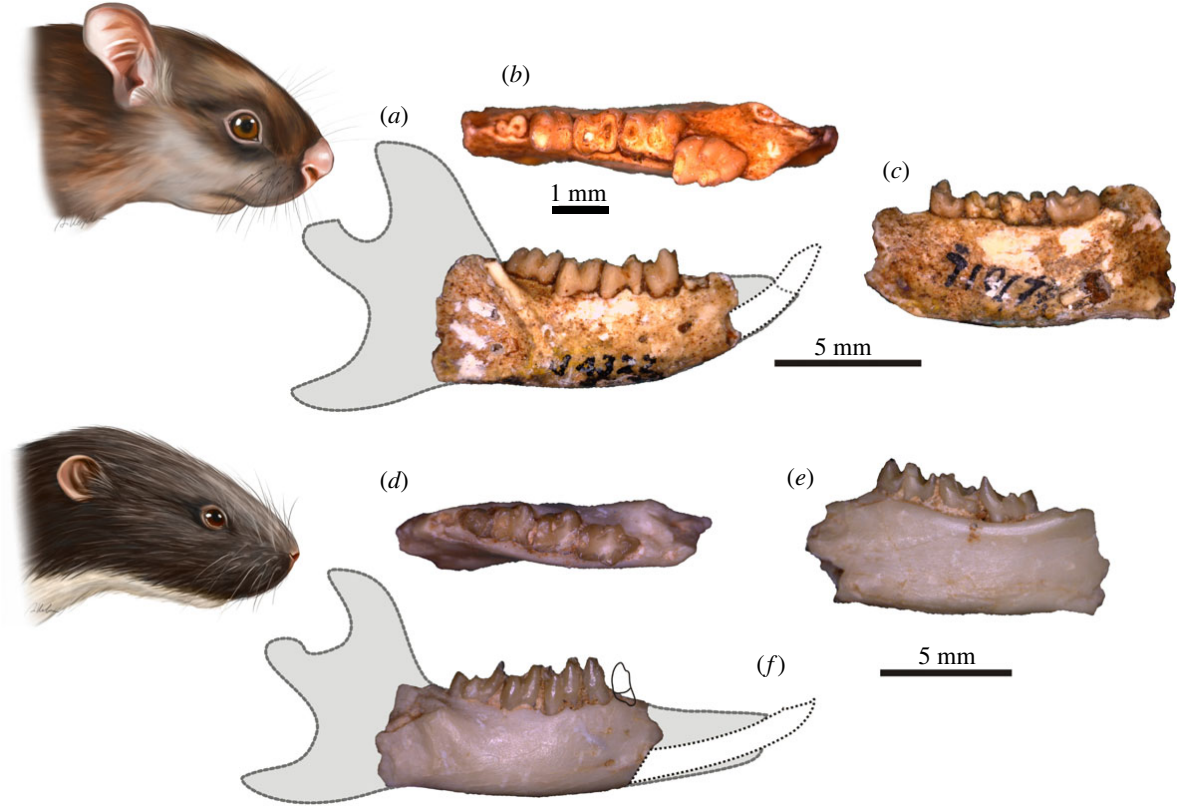
Mandible morphology in eurymylid representatives of simplicidentate Glires. (*a–c*) *Heomys orientalis* (IVPP V 4321) in buccal, occlusal and lingual views; Qianshan locality, Anhui Province, China. (*d–f*) *Eomylus* sp. (IVPP V 25372) in occlusal, lingual, and buccal views; Nuhetingboerhe locality, Nei Mongol, China. Head visualizations by Agnieszka Kapuścińska.

We based our reconstructions of the mandibles of *Eomylus*, *Heomys* and both *Mimotona* species (figures 1 and 2) on the most complete Palaeocene and Eocene material of closely related taxa of mimotonids and eurymylids [2,11,13,15,36] as well as on the morphological data from the fossil (Ischyromyidae) and extant rodent taxa from the ‘Sciuromorpha’ suborder (*sensu* [42], i.e. aplodontids, sciurids and dormice), the group often considered to branch first within crown rodents (see [2,43,44]).

The extant comparative material used in the analysis of mechanical properties of the mandibles included: five rodents (hazel dormouse, *Muscardinus avellanarius*; Nagtglas's African dormouse, *Graphiurus nagtglasii*; eastern grey squirrel, *Sciurus carolinensis*; giant flying squirrel, *Petaurista* sp.; and mountain beaver, *Aplodontia rufa*) and two lagomorphs (European rabbit, *Oryctolagus cuniculus* and American pika, *Ochotona princeps*, a leporid and ochotonid, respectively). It should be noted that although the rodents all belong to the Sciuromorpha suborder, they exhibit diverse zygomasseteric morphotypes: *Sciurus* and *Petarurista* are sciuromorphous; *Graphiurus* is hystricomorphous; *Muscardinus* is pseudomyomorphous; and *Aplodontia* is protrogomorphous [34].

### (b) CT-scanning and data processing

**Figure 3.**
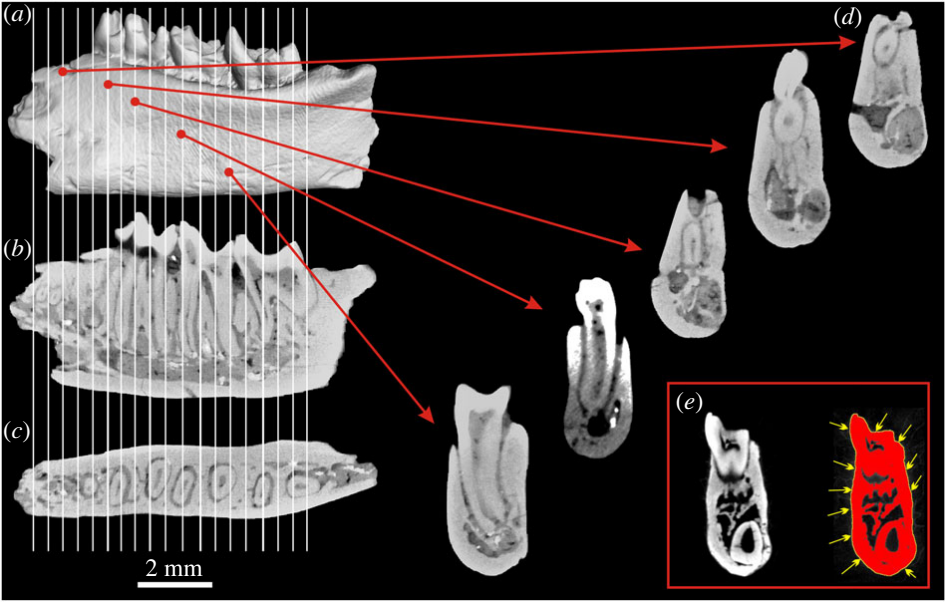
Cross-section sampling of the dentary for the analysis of mechanical properties. CT-scanned dentary of *Eomylus* sp. in lingual view (*a*); sections in the sagittal (*b*) and horizontal (*c*) plane showing root arrangements. White lines represent sampling sections of the cheek teeth (p4–m3) segment analysed for the section moduli and moment of inertia. Representative sections for the posterior portion of the incisor root and premolar-molar segments are shown at the right (*d*). An 8-bit cross-sectional microCT slice through the dentary of *Heomys* (*e* at the left), and the same slice with a threshold of 87–255 applied (*e*, at the right); section moduli and polar moment of inertia calculated from yellow outline (marked with arrows).

The Palaeocene Glires specimens from the collection of IVPP CAS, China were scanned using the 225 kV microCT scanner developed by the Institute of High Energy Physics, Chinese Academy of Sciences (CAS) at the Key Laboratory of Vertebrate Evolution and Human Origins, CAS. The specimens of Palaeocene Glires were scanned with beam energy of 120 kV and current of 120 mA at a resolution of 12.55 µm per voxel using a 360° rotation with a step size of 0.5°. A total of 720 projections were reconstructed in a 2048 × 2048 matrix of 1536 slices using a two-dimensional reconstruction software developed by the Institute of High Energy Physics, CAS. MicroCT scans of all specimens of extant taxa were downloaded from the Morphosource online repository (www.morphosource.org) and had voxel sizes ranging between 0.015 and 0.070 mm. Further details of the specimens, including links to Morphosource, are given in electronic supplementary material, table S1. Using Avizo v. 2020.3 (Thermo Fisher Scientific, Waltham, MA), each microCT scan was reoriented to align the long axis of the dentary along the *z*-axis, the dorsoventral axis along the *y* axis, and the mediolateral axis along the *x* axis. To correct for size differences and to allow the direct comparison of biomechanical measures from all the specimens, each scan was rescaled to an equivalent tooth row length (fourth premolar (p4) to third molar (m3) inclusive; figure 3). Scans were then imported into Fiji v2.35 [45], and the plugin MomentMacro v1.4B ([46]; https://fae.johnshopkins.edu/chris-ruff/) was used to calculate cross-sectional biomechanical measures for each of 19 equally spaced slices spanning the p4–m3 tooth row section, and also at the midpoint along the mesio-distal axis of each tooth. Owing to post-mortem damage, these could only be calculated for the first 14 slices of *M. lii* and *M. robusta*. Following Adams *et al*. [30], significant differences in the biomechanical measures between the fossil Glires and other specimens were tested with paired Wilcoxon signed-rank tests using PAST v4.11 [47]. Given the large number of pairwise comparisons, a Bonferroni correction was made to the *p*-values to account for the increased probability of Type I errors.

### (c) Cross-sectional geometry

To gain an understanding of how the fossil dentaries would have performed biomechanically during mastication, we derived three biomechanical measures from the cross-sectional morphology of the lower jaws: section modulus in the dorsoventral (*Z_x_*) and mediolateral (*Z_y_*) planes, and the polar moment of inertia (*J*). The section moduli represent the ability of the dentary to resist bending in the specified axis and are calculated by dividing the second moment of area by the distance from the neutral axis to the outer edge in the plane of bending [26]. The polar moment of inertia is calculated by summing the dorsoventral and mediolateral second moments of area and represents the ability of the dentary to resist torsion around its long axis.

## 3. Mandible morphology

Rodent mandibles show great morphological diversity owing to variability in the diastema length, the shape and depth of the mandibular body and the position of the condylar process. Also, the deviation of the angular process from the mandible body plane is related to two main types of mandibular structure, hystricognathous and sciurognathous [18,34]. By contrast, mandible morphology in lagomorphs is quite uniform and generally much simpler, with inter-generic differences concerning mostly the body depth and relative diastema length. All dentary structures in Lagomorpha, from the anteriormost tip to the end of the angular process, are coplanar, and the main differences between the two extant families (Leporidae and Ochotonidae) are in the shape of the angular process, which in ochotonids forms an acute posterior spur, whereas in leporids it is well rounded [48]. From a morphological standpoint, the dentaries of stem Glires, especially the Palaeocene representatives (figures 1 and 2), are similar, at least regarding the mandibular body structure (see e.g. [12,15,35]). Overall, the mandibles are much more like the lagomorph morphotype (compare [36,49]) than the rodent morphotype, even in Rodentiaformes such as *Tribosphenomys* [17].

The dentaries of the stem Glires are relatively slim to moderately deep with a ratio of tooth row length versus maximum mandible depth between 2 and 1.75. The ventral margin of the dentary is either almost straight as in *Eomylus* sp. (figure 2) or gently curved as in *Heomys orientalis* or *Mimotona lii* (figures 1 and 2). Only rarely do some species, such as *Mimotona robusta* (figure 1) or *Hanomys* (see [50]), show a greater deepening of the mandible body, with ratio of dental row to the dentary depth 1.47 and 1.62, respectively. The point of the maximum depth of the dentary lies mostly between the m1 and m2 alveoli. Unlike later genera, the diastema is not very elongated in stem Glires; in most species it is rather short. The exceptions are some Eocene taxa such as *Rhombomylus* having a more elongated diastema [2] and the late Palaeocene eurymylid from Mongolia, *Khaychina*, which has a markedly elongated diastema [15].

The most complete fragments of the angular process among Palaeocene simplicidentates are in the eurymylid *Hanomys* [50] and the rodentiaform *Tribosphenomys* [17]. The remains of the angular process in these taxa are suggestive of a pronounced acute posterior spur, typical of most Glires, including stem lagomorphs ([41]: figure 2*e,f*), extant ochotonids [48], and most rodent lineages (see electronic supplementary material). The exceptions displaying the rounded or reduced angular process can be found in leporids [48] and some specialized rodents with shortened and strongly deepened mandibles such as *Pedetes* (see electronic supplementary material), which are clearly derived. Moreover, a posteriorly acute angular process seems to represent a morphology frequently seen in the stem placentals (see e.g. [32,51]).

A complete condylar process is known only in the Eocene Glires taxa, and in both *Rhombomylus* and *Gomphos* it is distinct, large and extended dorsally, forming a substantial vertical ramus of the mandible with a well-developed coronoid process, usually gently hooked posteriorly and higher than the mandibular condyle [11,36]. In extant Glires, the shape and size of the coronoid process are very variable, from moderately large to almost non-existing; the latter state is typical of lagomorphs (see [41,48]). The orientation of the vertical ramus of the mandible also differs among Glires, with lagomorphs showing an apparently ancestral character of a strongly vertical condylar process and most rodents expressing posteriorly inclined and lower vertical rami. To sum up: the general morphology of the mandible in stem Glires resembles more closely lagomorphs in that all the processes of the mandible are arranged in one plane, with the mandibular body being relatively slim, the angular process forming a posteriorly extended spur, and the large condylar process being arranged more vertically.

## 4. Beam mechanics

**Figure 4.**
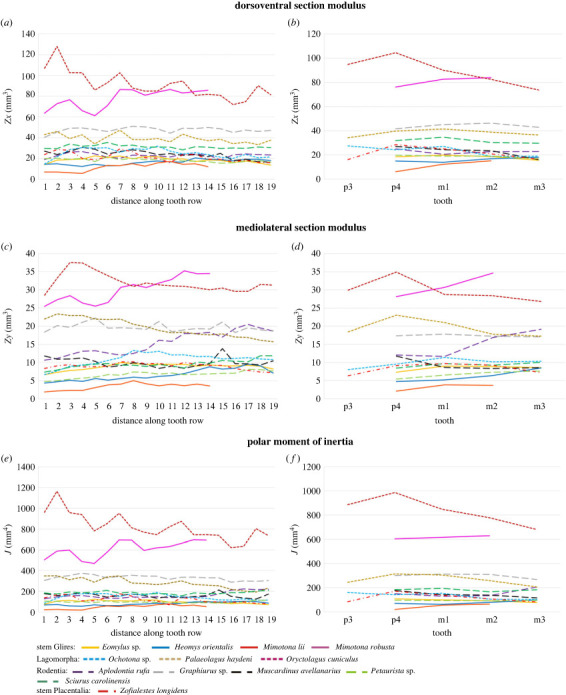
Plots of biomechanical analyses of all specimens in this study. (*a*,*b*) Dorsoventral section modulus, (*c*,*d*) mediolateral section modulus and (*e*,*f*) polar moment of inertia along molar tooth row. Dentaries scaled to the same p4–m3 length. (*a*,*c*,*e*) Numbers 1–19 represent slices taken at equidistant intervals along the dentary from anterior to posterior; (*b*,*d*,*f*) slices taken at midpoint of each tooth from p3 to m3 (not all teeth present in all specimens).

The plots of *Z_x_*, *Z_y_* and *J* for each species are given in figure 4 and electronic supplementary material, figure S3. The biomechanical values are presented in two ways: at regular intervals along the tooth row (to provide a more granular visualization of change along the dentary; figure 4*a*,*c*,*e*), and also at the midpoint of each tooth (to allow comparisons at specific teeth between specimens; figure 4*b*,*d*,*f*). Among stem Glires, *Eomylus* does not show any pronounced changes in the dentary resistance to torsion and bending along the dental row; however, both section moduli are slightly higher in the anterior half of the tooth row than the posterior (especially at m1; figure 4*b*,*d*), indicating that it is most resistant to bending in this region. The polar moment of inertia does not show any visible pattern, indicating that the resistance to torsion was generally similar along the tooth row, perhaps slightly stronger in the anterior part of the dentary (see electronic supplementary material). By contrast, *Heomys* shows an increase in all three measures moving posteriorly along the tooth row (especially posteriorly to m1; figure 4*a–d*), although the change in resistance to the dorsoventral and mediolateral bending is more notable than the resistance to torsion (polar moment of inertia), which is more uniform along the whole measured distance. These parameters demonstrate that the dentary is strongest in the region of the second and third molars. The mechanics of *Mimotona lii* was reconstructed only partially owing to the damage to its postero-ventral part. The studied part includes the dental row between p4 and m2. It exhibits a similar pattern to *Heomys* due to dorsoventral bending, and polar moment of inertia, but the mediolateral modulus pattern follows that of *Eomylus* sp., with a somewhat marked peak around m1 (figure 4*c*). The general observation is that the posterior part of the dentary is stronger. *Mimotona robusta* is notable for having much greater values of *Z_x_*, *Z_y_* and *J* than other stem Glires, despite the scaling of the specimens. This reflects the relatively increased depth of this dentary. The pattern along the dentary indicates that *M. robusta* is strongest toward the middle and posterior part of the tooth row in all three axes, although the polar moment of inertia is the least affected by the change.

The data obtained for stem Glires show patterns different from those of the other species. The resistance to dorsoventral bending is similar across the studied lagomorphs, with a more or less strongly expressed posterior decrease in mandible strength along the tooth row (figure 4*a*,*b*), differing only in the location of the maximum strength values. In *Oryctolagus* it falls at p4, whereas in *Ochotona* and in *Palaeolagus* it is at m1. *Ochotona* differs from *Palaeolagus* in having also a strengthened region near the p3 alveolus, whereas the *Palaeolagus* pattern is more consistent with the pattern observed for *Eomylus*, yet different from other stem Glires taxa, which show the reverse pattern of a posteriorly increased resistance to dorsoventral bending (figure 4*a*,*b*). In extant rodents, the changes in dorsoventral modulus are not very significant, but different from lagomorphs, *Heomys* and *Mimotona*. Three slightly different patterns can be observed for rodents: a slight posterior strengthening of the mandible in *Graphiurus*, a posterior weakening of the mandible in *Muscardinus* and *Sciurus*, and generally constant values along the whole dental row in *Aplodontia* and *Petaurista*. The stem placental, *Zofialestes*, exhibits a similar pattern to *Oryctolagus*, with peak in *Z_x_* around m1 followed by reduction in dorsoventral strength.

The section modulus in the mediolateral aspect shows more variable patterns across the studied taxa (figure 4*c*,*d*). An increase of the values posteriorly along the tooth row is observed in mountain beaver (*Aplodontia*) and sciurids (*Petaurista* and *Sciurus*). This pattern is consistent with *Mimotona* (*M*. *robusta* in particular) and *Heomys*. On the other hand, lagomorphs, especially *Oryctolagus* and *Palaeolagus*, (as well as dormice to a lesser extent) show a reverse pattern of posterior decrease in modulus, with the peak of the values observed at the p4 locus (figure 4*d*), whereas *Ochotona* has the peak shifted to m1 and thus displays higher modulus values in the mid-length of the tooth row, a feature shared with *Eomylus* and the stem placental, *Zofialestes* (figure 4*c*,*d*).

Resistance to torsion decreases posteriorly in all lagomorphs more profoundly than in *Eomylus*, whereas it increases posteriorly in *Aplodontia* and *Sciurus* (figure 4*e*,*f*), similar to *Heomys*, and both *Mimotona* species. In *Graphiurus*, *Muscardinus* and *Petaurista* it shows general oscillations along the tooth row, dropping slightly posteriorly (especially posteriorly to m2).

The data for *Zofialestes* are consistent with trends observed for Lagomorpha. The dorsoventral modulus pattern follows a general trend to decrease of the values posteriorly, along the tooth row, observed in all studied lagomorph taxa. The mediolateral moduli peak in the middle of the tooth row length, similar to *Ochotona*, whereas the values of the polar moment of inertia in *Zofialestes* decrease posteriorly along the tooth row, as in *Oryctolagus* and *Palaeolagus*.

The paired Wilcoxon signed-rank tests (table 1) indicate that, even with Bonferroni corrections, the fossil dentaries are significantly different in their bending and torsional strength from most of the other comparator species studied here, although with some notable exceptions. *Eomylus* is not significantly different from *Petaurista* in *Z_x_* and *J*, and from *Muscardinus* and *Zofialestes* in *Z_y_* (table 1). *Heomys* is not significantly different from *Petaurista* in *Z_x_* and *Z_y_*. After Bonferroni correction, *Zofialestes* is also not significantly different from both *Eomylus* and *Heomys* in *Z_x_* and *J*. *Mimotona lii* is significantly different from all other specimens, and *M. robusta* is highly significantly different from all other specimens except *Oryctolagus* in mediolateral bending strength (*Z_y_*). However, it should be noted that only the first 14 values of the parameters could be compared owing to the damage of the *Mimotona* specimens.

**Table 1.** Comparative *p* values (A) and Wilcoxon test values (B) of section modulus in the dorsoventral (*Z_x_*) and mediolateral (*Z_y_*) planes, and the polar moment of inertia (*J*) in analysed taxa; number of asterisks marks the statistical significance strength (from the highest (***) to the lowest (*)); n.s., not significant.

	*Eomylus*		*Heomys*		*Mimotona lii*		*Mimotona robusta*	
	A	B	A	B	A	B	A	B
*Z_x_*								
*Aplodontia*	0.00034	184***	0.00016	189***	0.00098	105***	0.00098	105***
*Graphiurus*	0.00013	190***	0.00013	190***	0.00098	105***	0.00098	105***
*Muscardinus*	0.00013	190***	0.0004	183***	0.00098	105***	0.00098	105***
*Petaurista*	0.65801	106 n.s.	0.05341	143 n.s.	0.00098	105***	0.00098	105***
*Sciurus*	0.00013	190***	0.00013	190***	0.00098	105***	0.00098	105***
*Ochotona*	0.00013	190***	0.00013	190***	0.00098	105***	0.00098	105***
*Oryctolagus*	0.00013	190***	0.00013	190***	0.00098	105***	0.00429	98***
*Palaeolagus*	0.00013	190***	0.00013	190***	0.00098	105***	0.00098	105***
*Zofialestes*	0.02582	150*	0.00549	164**	0.00123	104**	0.00098	105**
*Z_y_*								
*Aplodontia*	0.00013	190***	0.00013	190***	0.00098	105***	0.00098	105***
*Graphiurus*	0.00013	190***	0.00013	190***	0.00098	105***	0.0098	105***
*Muscardinus*	0.05341	143 n.s.	0.00013	190***	0.00098	105***	0.00098	105***
*Petaurista*	0.00013	190***	0.7475	103 n.s.	0.00098	105***	0.00098	105***
*Sciurus*	0.01001	159**	0.00013	190***	0.00098	105***	0.00098	105***
*Ochotona*	0.00013	190***	0.00013	190***	0.00098	105***	0.00098	105***
*Oryctolagus*	0.00013	190***	0.00013	190***	0.00098	105***	0.19812	76 n.s.
*Palaeolagus*	0.00013	190***	0.00013	190***	0.00098	105***	0.00098	105***
*Zofialestes*	0.33414	119 n.s*.*	0.00116	176**	0.00098	105***	0.00098	105***
*J*								
*Aplodontia*	0.00013	190***	0.00013	190***	0.00098	105***	0.00098	105***
*Graphiurus*	0.00013	190***	0.00013	190***	0.00098	105***	0.00098	105***
*Muscardinus*	0.00013	190***	0.00013	190***	0.00098	105***	0.00098	105***
*Petaurista*	0.39806	116 n.s.	0.00376	162**	0.00098	105***	0.00098	105***
*Sciurus*	0.00013	190***	0.00013	190***	0.00098	105***	0.00098	105***
*Ochotona*	0.00013	190***	0.00013	190***	0.00098	105***	0.00098	105***
*Oryctolagus*	0.00013	190***	0.00013	190***	0.00098	105***	0.00098	105***
*Palaeolagus*	0.00013	190***	0.00013	190***	0.00098	105***	0.00098	105***
*Zofialestes*	0.0126	157*	0.0621	163**	0.00123	104**	0.00098	105***

## 5. Discussion

The exact biomechanical adaptations related to mastication and bite force in the Palaeocene Glires have previously been unstudied, mostly owing to the incomplete preservation of the cranial and mandibular material that would enable a functional analysis based on muscle attachments and forces acting during chewing. For the first time, we used beam mechanics to study the mandibular corpus resistance to bending and torsion, which allowed us to analyse partially preserved dentaries of the early Glires.

Our results reveal that the early stem Glires were already diversified and specialized in their chewing modes, which implies different dietary specializations, and thus niche partitioning for this group already in the early Palaeocene. The overall mandible morphology of the examined taxa agrees more closely with the lagomorph morphotype, although the data are limited mostly to the mandible bodies and associated dentition. Regarding the biomechanical properties, even taxa with a double incisor set, which are thus considered duplicidentate, and as such are supposedly closer to the lagomorphs, do not express typically lagomorph patterns of resistance to bending and torsion (figure 4). *Mimotona robusta*, a duplicidentate taxon showing a deep mandible body, displays a mediolateral section modulus value not significantly different from that of *Oryctolagus*, but its overall patterns in bending and torsion resistance along the cheek teeth row are more typical of mountain beaver and sciurids (figure 4), and similar to *Ochotona* only in the anterior half of the mandible (figure 4*a*,*b*). The same can be said in general of *Mimotona lii*, which displays similar patterns in mechanical resistance to bending and torsion (albeit at a much lower magnitude). In general, *Heomys orientalis* and both *Mimotona* species show the dentary strengthening posteriorly, which may imply some prevalence of grinding force over gnawing [30], although the latter factor was not analysed in this study, nor for other stem Glires. In fact, the anterior part of the skull of *Heomys* bears very powerful upper incisors, strongly recurved posteriorly, similar to those of sciurids, which suggests an excellent gnawing ability. Furthermore, the high and flat lateral aspect of the muzzle in *Heomys* with a marked horizontal ridge could point to the possibility of the sciuromorphous type of the anterior deep masseter attachment [52]. The sciuromorphous masseteric condition has been noted to be more efficient in incisor biting than the supposedly ancestral protrogomorphous condition [3,53], and has recently been suggested to have evolved much earlier within Rodentia than previously suspected [54]. The presence of a truly sciuromorphous masseter type in the stem Glires would cast a new light on the rodent ancestral morphotype and the extent of convergent masseter morphology among Glires taxa [43].

The above-mentioned rodent-like pattern of the mandible mechanical properties displayed by *Heomys* and *Mimotona* contrasts with that of *Eomylus*. The latter shows posteriorly decreasing resistance to dorsoventral bending as well as to torsion or generally uniform resistance to mediolateral bending, along the whole dentary length. Thus, the most powerful segment of *Eomylus* dentary falls to the premolar (and m1) portion, the pattern shown also by the analysed lagomorph taxa. The pattern of resistance to dorsoventral and mediolateral bending as well as to torsion in *Eomylus* agrees also with that for *Zofialestes*. This biomechanical information suggests a quite different diet in *Eomylus* compared with *Heomys* and *Mimotona*. The latter were apparently more adapted to harder food, such as grains and nuts, whereas *Eomylus* expressed adaptations to a much softer diet likely composed of fruits, leaves or possibly soft-bodied insects.

Regarding the ancestral Glires mandibular morphotype, the late Palaeocene *Eomylus*, given its similarities to *Zofialestes*, appears to provide a better candidate than the much earlier *Heomys* or *Mimotona*. In agreement with the prevailing view, convergence and parallelism are widespread phenomena in early Glires evolution [5], as well as in Euarchontoglires at large (see [20]), showing the need for further phylogenetically informative characters from later Mesozoic/earliest Palaeocene mammals to produce a clearer understanding of the earliest rodent and lagomorph ancestral morphotypes.

## Data Availability

Virtual reconstructions of the four Palaeocene Glires dentaries are available from the Dryad Digital Repository: https://dx.doi.org/10.5061/dryad.69p8cz95g [55]. MicroCT scans of the extant taxa are available for download from Morphosource (www.morphosource.org). Links are provided in electronic supplementary material, table S1. Additional data are provided in the electronic supplementary material [56].
